# Functional fitness, lifestyle and demographic factors as predictors of perceived physical and mental health in older adults: A structural equation model

**DOI:** 10.1371/journal.pone.0290258

**Published:** 2023-09-06

**Authors:** Giovanni Angelo Navarra, Garden Tabacchi, Antonino Scardina, Massimiliano Agnese, Ewan Thomas, Antonino Bianco, Antonio Palma, Marianna Bellafiore

**Affiliations:** Sport and Exercise Sciences Research Unit, Department of Psychology, Educational Science and Human Movement, University of Palermo, Palermo, Italy; University of Illinois at Urbana-Champaign, UNITED STATES

## Abstract

Over the past 50 years, socioeconomic development has brought a reduction in birth rates, an increase in life expectancy and consequently in the elderly population. For this reason, there has been an increasing focus on physical and mental health of the elderly, promoting the concept of healthy aging. The aim of this study was to explore the associations between perceived physical and mental health of older adults and a variety of determinants, such as demographic factors, physical functional fitness, physical activity level, adherence to the Mediterranean diet and anthropometric indices, through a structural equation modeling (SEM). A cross-sectional observational study involved 208 elderly (24 men and 184 women) over the age of 60, fully independent and autonomous. Perceived physical and mental health were assessed with the Short Form 12 questionnaire. Basic sociodemographic information was collected; anthropometric features were directly measured, functional fitness was assessed with the Senior Fitness Test, and physical activity level was determined through the International Physical Activity Questionnaire; adherence to Mediterranean Diet (MD) was also collected through the MEDAS questionnaire. The SEM analysis revealed that functional fitness, which was a latent variable of the model described by the six administered fitness tests, was a strong predictor both of perceived physical and perceived mental health in the sample of elderly. Physical activity level was as predictor of the perceived physical component, but not of the mental health, while score of metabolic equivalent task did not result a predictor, as well as the sociodemographic factors and adherence to MD. The present findings suggest that it would be strongly recommended for elderly subjects to engage in physical activity specifically targeted to aged populations, in order to enhance their fitness abilities and enable them to improve the perception of their own health status.

## Introduction

Over the past 50 years, globally, socioeconomic development has led to a reduction in new births and an increase of life expectancy [1]. This trend has inevitably caused changes in population demographics by favoring an increase in the elderly population over the young. So, the World Health Organization (WHO) has estimated that by 2050 the world’s population of people aged 60 years and older will double, and the number of persons over 80 years old is expected to triple between 2020 and 2050 to reach 426 million [2]. For these reasons, WHO over the past few years has been thinking of intervention policies in order to ensure a better life perspective and healthy aging for older people [3, 4].

Aging is a natural and irreversible process involving a reduction in the physical and cognitive functions of the human body and the probability of the onset of age-related diseases [5]. The decline in physical abilities is strongly related to the phenomenon of aging and can impair even normal daily activities such as climbing or descending stairs, getting up from a chair, or simply walking independently. For many elderly individuals, a minimal loss of functional demand to perform normal daily activities can evolve into a drastic decrease in quality of life and loss of autonomy [6]. Functional fitness, in fact, is defined as the physiological ability to perform normal daily activities safely, independently, and without excessive fatigue [7].

Aging in the elderly is often characterized by a decrease in cognitive function sometimes correlated with the presence of anxiety and depression disorders [8, 9]. It is equally important for healthy aging, therefore, to consider not only the physical component but also the mental component and the relationship between these two.

Several studies have also shown that there is a strong association between physical and mental health [10, 11], between physical and psychological health [12], and between physical and cognitive function [13]. Ohrenberger’s study [10] have seen how physical and mental health depend on each other; in fact, a better past mental health significantly increases current physical health and better past physical health has a greater effect on current mental health. Similar is the study by Sha Luo et al. [11], which showed how physical health was closely related to changes in mental health and vice versa.

Healthy aging is a multidimensional concept closely related to a healthy lifestyle characterized by the performance of regular physical activity and healthy eating habits in order to preserve physical, social, mental health and well-being and quality of life [14]. Regular physical activity has been widely shown to be a protective factor in the prevention and treatment of the most common diseases that affect the elderly such as heart disease, stroke, diabetes, and some types of cancer [15]. In addition, the role of physical activity is also closely linked to the prevention of other important risk factors such as hypertension, overweight and obesity, and to improved mental health by delaying the onset of dementia and improving quality of life and general well-being [16, 17].

Promoting healthy aging may also depend on dietary modification, which can help prevent the onset of age-related diseases and effectively influence their development, thereby improving quality of life [18]. Specifically, it was noted that among various dietary patterns, high adherence to the Mediterranean Diet (MD) was associated with physical performance and cognitive function in the elderly [19, 20].

In the literature, the perception of the physical and mental state of the elderly can be influenced by several factors such as the absence of chronic diseases, but also by the degree of functional impairment, personality factors, stress level, and various psychosocial factors, as shown in the study of Knapik [21].

Although the determinants of healthy lifestyle are well known, little is known in the literature about how they interact with each other in order to predict physical and mental health in older people [22, 23].

The aim of this study was to explore the associations between perceived physical and mental health of older adults and a variety of determinants, such as demographic factors, physical functional fitness, physical activity level, adherence to the MD and anthropometric indices, through a Structural Equation Modeling (SEM). This statistical technique is widely used in the literature to modeling the interactions between variables by combining factor analysis and multiple regression analysis, while simultaneously accounting for measurement error [24]. The theoretical framework of the SEM is based on the path relationship between variables, with the abstract latent variables measured indirectly by the observed variables. This analysis tests first the measurement theory to confirm the reliability and validity of the measurement models; and after the measurement models are confirmed, the structural theory is tested to show how the latent variables are related to one another. Thus, SEM was used in the present study to provide a flexible framework for developing and clarifying the relationships among multiple observed and latent healthy lifestyle variables and perceived physical and mental health. To confirm the hypothesis of the relationship between the set of considered observed variables and their underlying latent constructs, a Confirmatory Factor Analysis (CFA) was performed before the SEM. Even though it is generally recommended to conduct an Exploratory Factor Analysis (EFA) before CFA, in the present study the researchers retained not necessary to use it, since already having a plausible hypothesis regarding the factor structure, i.e. the observed variables of the fitness tests likely explained a latent construct such as the “physical fitness” [25].

The advantages to the use of SEM are several: it allows observing complex and multidimensional models; the tested relations among factors are theoretically free of measurement error because the error has been estimated and removed, leaving only common variance; the measurement reliability can be accounted for explicitly within the analysis by estimating and removing the measurement error [24].

## Materials and methods

### Study design and participants

A cross-sectional observational study was conducted from November 2022 to January 2023, as part of an intervention evaluation within the Physical Activity Promotion & Domestic Accidents Prevention (PAP & DAP) Project, in collaboration with the Azienda Sanitaria Provinciale (ASP) of Palermo.

This study involved 208 elderly (24 men and 184 women) over the age of 60, fully independent and autonomous, with no physical and/or mental disabilities and cognitive disorders, from the city of Palermo and its Province. Participants were recruited from September to November 2022 through the ASP contact persons in the various districts of Palermo using posters with adherence on a voluntary basis.

Initially, there were 208 participants in the study, but due to various reasons (COVID-19, inability to perform some senior fitness tests or incorrect compilation of the questionnaires) we had fewer participants who completed the questionnaires or performed the Senior Fitness Test (SFT). SFT was performed by 154 participants, while SF-12, MEDAS, and IPAQ questionnaires were carried out by 126, 110, and 98 subjects respectively.

All participants gave their informed consent for the participation to the study, which was approved by the Ethical Board of the University of Palermo (N. 111/2022) and conformed to criteria for the use of persons in research as defined in the Declaration of Helsinki. All older adults participated voluntarily and could withdraw from the study at any time.

### Variables and measurements

Participants’ demographic information, i.e. age, gender, living area (city center, residential suburban, popular suburban), and anthropometric measures of weight and height were collected through the completion of a medical history card. BMI calculation is based on the following formula: weight in kilograms divided by height in meters squared. BMI was calculated to classify subjects according to weight status in underweight (below 18.5), normal weight (18.5–24.9), overweight (25.0–29.9), obese (30.0 and above) [26]. Several both qualitative and quantitative variables were then assessed through the administration of different questionnaires. Authors had access to information that could identify individual participants during data collection.

The Short Form 12 (SF-12) [27–29] is a questionnaire consisting of 12 items, taken from the 36 items in the original SF-36 questionnaire, that produce two measures related to two different aspects of health: perceived physical component and perceived mental component. The SF-12 consists of 4 scales: physical functioning, role and physical health, role and emotional state, and mental health. SF-12 questionnaire allows the description of health status through two summary indices calculated from the twelve questions: the index called Physical Component Summary (PCS) which concerns the physical health of the group, and the Mental Component Summary (MCS) which measures mental health. The results of the two indices PCS and MCS were evaluated through the algorithms in the spreadsheet for the *Microsoft Excel software* [30] (retrieved from http://amsacta.unibo.it/5751/). These two indices were taken as the dependent variables in our modeling study.

Participants’ functional fitness was assessed through the Senior Fitness Test (SFT) [31–33], a battery consisting of the following motor tests targeted to the population aged over 60: 1) Chair stand test, to test leg strength and endurance; 2) Arm curl in 30s, to test upper body strength; 3) 6 Minute Walking Test, to test aerobic capacity and endurance; 4) 2-minute step test, to test aerobic capacity and endurance; 5) Chair sit and reach, to measure the flexibility of the lower back and hamstring muscles; 6) Back stretch test, to assess flexibility in the shoulder joint and shoulder arch on the right and on the left side; 7) Timed up and go test, to assess mobility, balance, walking ability, and fall risk in older adults. The 6 Minute Walking Test was not administered because it is not always feasible in such aged population, as suggested by Langhammer et al. [34]; thus, aerobic capacity and endurance were evaluated only through the 2-minute step, which is better affordable by elderly people.

The physical activity (PA) level of the participants was assessed through the International Physical Activity Questionnaire (IPAQ) that consists of 7 items used to estimate the amount of physical activity performed in the past seven days by the subjects [35]. Participants are requested to report how many days and minutes of intense, moderate and walking activities they practice and how long they stay sedentary, in order to calculate a score corresponding to the metabolic equivalence task in minutes per week (MET min/week). It represents the amount of energy expended in performing physical activity. 1 MET is what you consume when you are at rest. For each type of physical activity, a different MET is obtained given by the multiplication between minutes engaged in physical activity, days, and a MET value arbitrarily assigned to each physical activity; walking is considered as 3.3 METs, moderate physical activity as 4 METs, and vigorous physical activity as 8 METs [36]. Thus, the final result is obtained through the sum of METs consumed for each type of physical activity (intense, moderate or walking). The patient will be classified as inactive if the total METs are less than 700, sufficiently active if the total is between 700 and 2519 and active or very active if the total is above 2520 METs [37, 38].

Mediterranean Diet Adherence Screener (MEDAS) is a 14-item questionnaire assessing adherence to the MD and major eating habits [39, 40]. Each response gives a score of 0 or 1 point, depending on whether or not the given response meets the criteria for adherence to the MD. As a result, the final MEDAS questionnaire score can range from 0 to 14. The higher the score, the better the adherence to the MD, which is considered acceptable when the score is greater than or equal to 9 [41, 42].

### Statistical analysis

The software STATA/MP 12.1 (Stata Corp, College Station, Texas) was used to analyse all data.

Quantitative variables used for the analyses included the following: the perceived physical health and perceived mental health measured as scores derived from the SF-12, which represented the dependent variables used in the structural model; BMI (expressed as Kg/m^2^), total MET min/week, MD adherence score, scores of the 6 tests of the SFT to evaluate functional fitness (upper and lower limb strength, flexibility of the lumbar and ischio-crural musculature, flexibility of the scapulohumeral joint, agility and dynamic balance, aerobic resistance). Qualitative variables were weight status (normal weight, overweight and obese), PA level (active or very active; fairly active; non active), MD adherence (yes/no), gender (M, F), living area (city center, residential suburban, popular suburban).

Normality of the distributions was tested through the Shapiro-Wilk test for normality.

Initially, bivariate correlation was performed to explore the associations between the variables and a correlation matrix was created to show them; Pearson’s coefficients were used for quantitative and normally distributed variables, while Spearman’s coefficients were used for categorical and not-normally distributed variables. Afterwards, confirmatory factor analysis (CFA) to modelling latent variables and their indicators was conducted.

Third, a structural equation model (SEM) was launched to examine predictors of physical and mental health. Both observed and latent variables were included in the model.

The following indices and cut-off were used to evaluate the goodness of the model: comparative fit index (CFI) and Tucker-Lewis Index (TLI), with values > = 0.9; root-mean square error of approximation (RMSEA), which tests the fit of the model to the covariance matrix, with value < = 0.06; the standardised root mean squared residual (SRMR), which is the square root of the discrepancy between the sample covariance matrix and the model covariance matrix, with an acceptable fit value of 0.08 or less. Akaike information criteria (AIC) and Bayesian information criteria (BIC) were also reported to compare the different models and choose the one with the smaller AIC and BIC (which is more likely to be replicated, has fewer parameter, and fits better) [43].

The examination of the standardized beta coefficients for hypothesized relationships was performed together with the standard errors. Statistical significance was set at p <0.05.

## Results

### Descriptive statistics

The descriptive analysis of quantitative variables (Table 1) shows that participants have a mean age of 71.2 (SD 5.63); they are generally slightly overweight (BMI >24.9), even though they are on average physically active with mean total MET >2591 (3503.4). They are not adherent to the MD on average, as their MD score is <9 (8.4).

**Table 1:** Quantitative characteristics of the sample.

	*N*	*Mean*	*SD* *
Age (year)	208	71.2	5.63
BMI (Kg/m^2^)	140	26.5	3.69
Perceived physical health score	126	43.6	8.30
Perceived mental health score	126	46.4	9.02
Chair stand test	152	10.5	2.56
Arm curl test	145	15.0	4.14
Sit and reach test	152	-7.8	9.37
Back scratch test	145	-10.2	15.25
Timed up-and-go test	154	8.3	2.18
2-minute step test	154	122.6	41.90
Total MET	98	3503.4	5062.36
MD^§^ score	110	8.4	2.01

*Standard Deviation

^§^Mediterranean Diet

The results of the fitness tests are showed both as scores (Table 1) and categories below average, on average and above average in Table 2.

**Table 2:** Fitness tests scoring of the sample by categories below average/average/above average.

	Chair stand test		Arm curl test		Sit and reach test		Back scratch test		Timed up-and-go test		2-minute step test	
	N	%	N	%	N	%	N	%	N	%	N	%
Below average	68	44.7	28	19.3	86	56.6	66	45.5	0	0	4	2.6
Average	83	54.6	86	59.3	66	43.4	59	40.7	16	10.4	47	30.5
Above average	1	0.7	31	21.4	0	0	20	13.8	138	89.6	103	66.9
Tot	152		145		152		145		154		154	

More than half participants have lower and upper limb strength in the average (54.6% and 59.3% respectively). The rest of the sample has lower limb strength below average (44.7%), while just one participant is above the average; for the upper limb strength, similar percentages are found both for values above the average (21.4%) and below (19.3%).

The flexibility of the lumbar and ischio-crural musculature estimated with the sit and reach test is below average for most participants (56.6%); the rest of the sample has it in the average.

The flexibility of the scapulohumeral joint is also below average for most participants (45.5%), but there is a small percentage (13.8%) of persons above the average.

The agility and dynamic balance revealed by the timed up-and-go test is for almost all sample (89.6%) above the average.

Finally, the aerobic capacity is above the average for most participants (66.9%), while only 2.6% is below the average.

Table 3 shows the frequencies of the considered qualitative variables. The sample is mostly represented by women (88.5%), coming mainly from residential suburban areas (54.5%). A total of 62.9% of participants is overweight/obese. The physical activity level is active/very active for a high percentage (49.0%). More than half sample (51.8%) does not show MD adherence.

**Table 3:** Qualitative characteristics of the sample.

*Gender*	*N*	*F*
M	24	11.5
F	184	88.5
Tot	208	
*Weight status*		
Normal weight	52	37.1
Overweight	63	45.0
Obese	25	17.9
Tot	140	
*Physical activity level*		
Active or very active	48	49.0
Fairly active	27	27.6
Non active	23	23.4
Tot	98	
*MD*^§^ *adherence*		
Yes	53	48.2
No	57	51.8
Tot	110	
*Living area*		
City center	44	28.2
Residential suburban	85	54.5
Popular suburban	27	17.3
Tot	156	

^§^Mediterranean Diet

### Bivariate correlation

The bivariate correlation between the study variables is shown in Table 4.

**Table 4:** Correlation matrix of the study variables. Pearson’s correlation coefficients were used for quantitative variables and normally distributed variables. Spearman’s correlation coefficients were used for categorical variables and not normally distributed variables.

	**Gender (ref.male)**	**Age**	**Living area (ref. central)**	**Chair stand test**	**Arm curl test**	**Sit and reach test**	**Back scratch dx test**	**Timed up-and-go test**	**2-minute step test**	**BMI (Kg/m** ^**2**^ **)**	**Weight status (ref. normal)**	**Total MET**	**Activity level (ref.inactive)**	**MD**^**§**^ **score**	**MD**^**§**^ **adherence (ref. non adherent)**	**Perceived physical health**	**Perceived mental health**
Gender (ref.male)	1.00																
Age (year)	0.23**	1.00															
Living area (ref. central)	-0.08	0.07	1.00														
Chair stand test	-0.06	-0.16^	-0.04	1.00													
Arm curl test	0.09	-0.24**	-0.14	0.54***	1.00												
Sit and reach test	0.19*	-0.10	0.01	0.25**	0.27**	1.00											
Back scratch test	0.18*	-0.13	-0.10	0.21*	0.30***	0.09	1.00										
Timed up-and-go test	0.06	0.27***	0.00	-0.70***	-0.56***	-0.28***	-0.17*	1.00									
2-minute step test	-0.20**	-0.17	-0.05	0.58***	0.51***	0.25**	0.14	-0.59***	1.00								
BMI (Kg/m^2^)	-0.06	-0.14	0.03	0.02	0.10	-0.04	-0.21*	-0.01	0.09	1.00							
Weight status (ref. normal weight)	0.09	-0.14	-0.15	0.00	0.01	0.00	0.01	0.02	-0.12	-0.05	1.00						
Total MET	0.02	-0.07	-0.13	0.02	0.23*	0.11	-0.22^	-0.21^	0.29*	-0.03	0.24^	1.00					
Physical activity level (ref. inactive)	0.07	-0.17	0.13	0.22^	0.23^	0.26*	-0.08	-0.39**	0.43***	0.01	-0.15	0.92***	1.00				
MD^§^ score	-0.13	-0.06	-0.13	0.10	-0.07	-0.23*	0.01	-0.04	0.17	-0.17^	-0.08	0.11	-0.12	1.00			
MD^§^ adherence (ref. non adherent)	-0.06	-0.05	-0.16	-0.08	0.03	0.11	0.02	0.11	-0.22*	0.14	-0.28*	0.26*	0.27*	-0.82***	1.00		
Perceived physical health	-0.01	-0.19*	-0.02	0.22*	0.20^	0.20^	-0.06	-0.34**	0.18^	-0.06	-0.02	0.24*	-0.37**	-0.15	-0.03	1.00	
Perceived mental health	-0.09	0.02	0.12	0.30**	0.35**	0.08	0.05	-0.36***	0.31**	-0.04	0.15	0.21^	0.14	-0.12	-0.05	0.12	1.00

The perceived physical health is significantly correlated to the agility and dynamic balance measured through the timed up-and-go test (r = -0.34, p<0.01), to the leg strength and endurance measured through the chair stand test (r = 0.22, p<0.05) and to the total MET and to the physical activity level (r = 0.24, p<0.05 and -0.37, p<0.01 respectively); a trend exists for the correlation with the upper limb strength and with the flexibility of the lumbar and ischio-crural muscles (r = 0.20, p<0.10 in both arm curl test and sit and reach test).

Perceived mental health is correlated to all tests of functional fitness (p<0.01), except sit and reach and back scratch; a trend was identified with Total MET (rho = 0.21, p<0.10).

Other correlations are evident for total MET with upper limb strength (r = 0.23, p<0.05) and aerobic resistance (r = 0.29, p<0.05); physical activity level with flexibility of the lumbar and ischio-crural musculature (r = 0.26, p<0.05), agility and dynamic balance (r = -0.39, p<0.01) and aerobic resistance (r = 0.43, p<0.001); weight status and MD adherence (rho = -0.28, p<0.05), with those adherents to the MD being less overweight or obese; physical activity level and MD adherence (rho = 0.27, p<0.05), with those adherents to the MD having a higher level of PA. A trend has been identified for Total MET and weight status (rho = -0.24, p<0.10), with people overweight/obese having a lower total MET.

### SEM model

A first model with three latent variables (i.e. functional fitness, physical condition, demographic factors) did not fit well and wasn’t considered. A second model was analyzed including two latent variables, i.e. functional fitness and physical general status, which was considered better but did not satisfied the cut-points of the indices (RMSEA 0.091, AIC 3905.414, BIC 3931.479, CFI 0.771, TLI 0.793, SRMR 0.152) [43].

**Fig 1:**
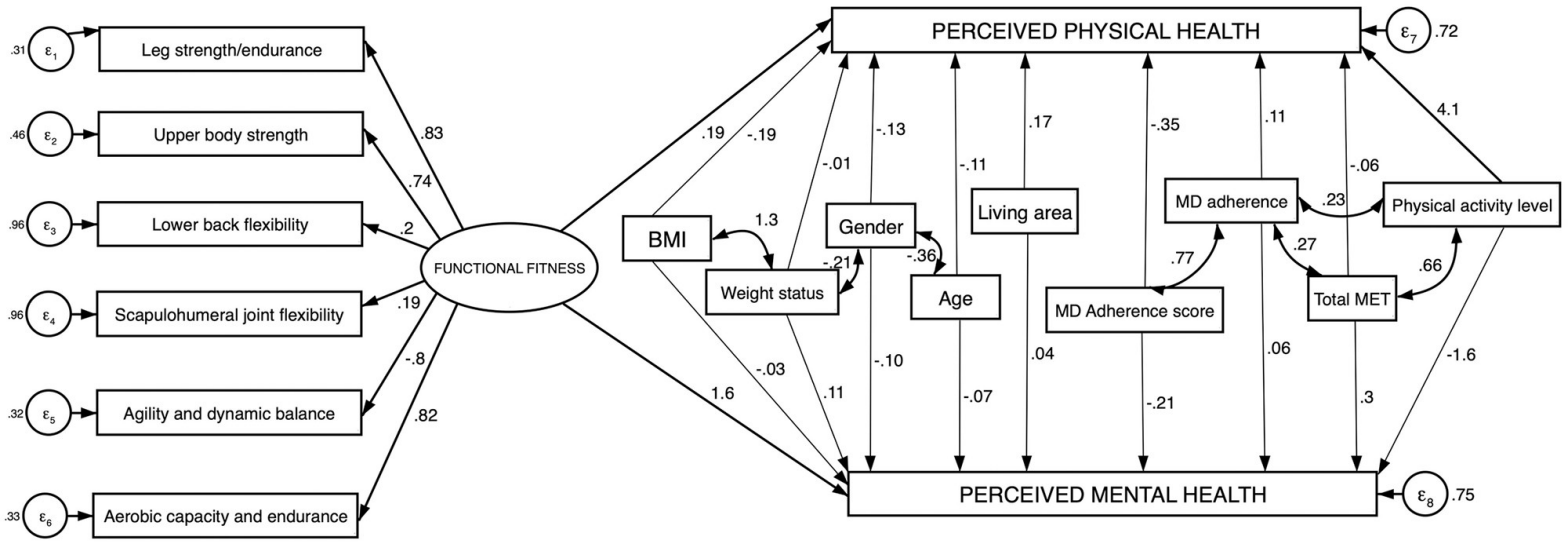
Final model predicting perceived physical and mental health in the considered sample. Relations are showed as standardized beta-coefficients. Thick lines represent significant relationships (p<0.05), two sided arrows represent covariances. Model fit indices: RMSA 0.028, AIC 3842.615, BIC 3866.942, CFI 0.958, TLI 0.963, SRMR 0.08.

The final model revealing the best fit (shown in Fig 1) included one latent variable, the functional fitness, and was described by the following indices: RMSA 0.028, AIC 3842.615, BIC 3866.942, CFI 0.958, TLI 0.963, SRMR 0.08.

Both physical and mental health are positively and significantly predicted by functional fitness. Structural and measurement models are also shown in Table 5, where non standardized beta-coefficients are showed. Those with better performances in the fitness tests have a physical status perception 0.2 points higher than those having worse performances; people showing higher functional fitness also have perception of their mental status 0.35 points higher.

**Table 5:** Structural and measurement models of the relationships between dependent variables (perceived physical and mental health) and different examined predictors included in the SEM analysis. Values are expressed as unstandardized beta-coefficients.

Structural model	Beta coefficient (raw)	SE	p	Lower CI (95%)	Upper CI (95%)
**Perceived physical health**					
Gender (ref.male)	0.02	0.088	0.870	-0.16	0.188
Age	-0.10	0.094	0.313	-0.28	0.09
Residence area (ref. central)	0.03	0.099	0.732	-0.16	0.23
BMI	-0.19	0.138	0.154	-0.45	0.07
Weight status (ref.normal weight)	0.10	0.138	0.440	-0.16	0.38
MD adherence score	-0.32	0.167	0.055	-0.65	0.01
MD adherence (ref.non adherent)	0.13	0.179	0.454	-0.22	0.48
Total MET	0.08	0.117	0.498	-0.15	0.31
Physical activity status (ref.non active)	0.27	0.127	**0.036**	0.02	0.52
Functional fitness	0.21	0.099	**0.037**	0.01	0.40
**Perceived mental health**					
Gender (ref.male)	-1.28	0.088	0.145	-0.30	0.04
Age	0.02	0.135	0.882	-0.24	0.29
Residence area	0.12	0.098	0.227	-0.07	0.31
BMI	-0.24	0.147	0.106	-0.53	0.05
Weight status (ref.normal weight)	0.21	0.136	0.131	-0.06	0.47
MD adherence score	-3.35	2.757	0.224	-8.75	2.05
MD adherence (ref.non adherent)	2.81	2.802	0.315	-2.68	8.31
Total MET	0.12	0.114	0.275	-0.10	0.35
Physical activity status (ref.non active)	-0.04	0.136	0.756	-0.31	0.22
Functional fitness	0.35	0.090	**0.000**	0.18	0.53
**Measurement model**					
**Functional fitness**					
Chair stand test	0.82	0.04	**0.000**	0.74	0.89
Arm curl test	0.70	0.05	**0.000**	0.60	0.80
Sit and reach dx test	0.33	0.08	**0.000**	0.18	0.49
Back scratch dx test	0.26	0.09	**0.003**	0.09	0.44
Timed up-and-go test	-0.83	0.04	**0.000**	-0.90	-0.76
2-minute step test	0.71	0.05	**0.000**	0.61	0.81

The physical activity level is only related to physical status: active people have a higher and better perception of their physical status of 0.3 points more compared to inactive people.

All the other considered variables were not predictors of perceived physical and mental health.

## Discussion

This study explored the relationship between perceived physical and mental health in older adults and several determinants, such as demographic factors, functional fitness, physical activity level, anthropometric indices and adherence to the Mediterranean diet. Correlations between the considered variables were described by an initial bivariate analysis, followed by a structural equation modeling that explained the predictors of perceived physical and mental health.

The final obtained model revealed one latent variable, the functional fitness, which was described by the six fitness abilities measured through the SFT and this was confirmed by the highly significant coefficients (Table 5) showed in the measurement model. This latent variable was significantly associated both with perceived physical and mental health, showing standardized coefficients respectively of 0.19 and 1.6.

Regarding the performances at the fitness tests, our sample generally showed quite good results (in the average or above the average) for the upper body strength, agility/dynamic balance and aerobic capacity/endurance. These values were significantly correlated in the bivariate analysis with the physical activity level, and this result is consistent with the study by Buchman et al. [44] who showed that physical activity was modestly associated with global motor function, motor performance and muscle strength in older people. Buchman also showed that people with a high level of physical activity had a slower rate of decline in motor function by more than 35% compared with those who did not engage in any form of physical activity and that there was no sex difference in the association between physical activity and change in motor function. This last finding mitigates the limitation of our sample which consisted mostly of women. Agility and dynamic balance, detected by timed up and go test, were for almost the entire sample above average, as well as aerobic capacity, assessed through the 2-minute step test, for most participants. This result is consistent because most of the participants’ self-reported physical activity through the IPAQ questionnaire was related to walking, which is the usual activity for performing most daily tasks.

With concern to the other fitness abilities, most of our sample showed that the flexibility of the lumbar and ischio-crural muscles estimated with the sit-and-reach test, as well as the flexibility of the scapulo-humeral joint were below average. This result could be attributed to the chronic use of these joints, which are the most stressed in the activities of daily living [45] and not to the participants’ physical activity levels. Stathokostas’ study also showed that there is no relationship between self-reported physical activity levels and upper or lower body flexibility. The study by Walker et al. [46] also reported no differences in ranges of motion of some body joints in a sample of 60 elderly men and women classified into high and low physical activity categories based on self-assessment. Miotto et al. [47] found no difference comparing flexibility of the posterior thigh muscles in a sample of active versus sedentary adults with an average age of 68 years. Physical activity level, therefore, did not explain a significant amount of the variance in flexibility measures, and flexibility was not associated with functional ability.

At the bivariate analysis we also found that perceived physical health was significantly correlated with agility and dynamic balance and total MET/physical activity level; for this reason, there is a trend (p<0.10) for correlation with lower and upper limb strength and with flexibility of lumbar and ischio-crural muscles.

On the other hand, a study by Pucci et al. showed that there is a positive association between physical activity and Short Form-36 questionnaire domains including "physical functioning", "vitality", "mental health", "role-physical", "role-emotional", "general health" and "physical and mental components". In particular, the domains "physical functioning", "vitality" and "mental health" showed a greater association of these domains with physical activity [48].

The structural model showed the perceived mental health being highly correlated with all tests of functional fitness. To our knowledge, there are not studies in the literature assessing this relationship, but our results can suggest that old people should gradually and constantly build and maintain adequate levels of fitness abilities (such as strength of upper and lower body, flexibility, agility, dynamic balance and aerobic capacity) in order to perceive a better health status. The bivariate analysis showed this correlation for all tests except the sit and reach and back scratch tests. This is consistent with the work of Hemmeter et al. that physical fitness counteracts the development of depression; in fact, physical fitness is associated with better mental health in the elderly [49]. Physical activity induces changes in brain neurotransmitters and endogenous opioids associated with depression, anxiety, and other mental health problems, improving the relationship between physical activity and quality of life [50]. A study by Byeon et al. found that elderly people who did flexibility exercises between 1 and 4 days had an approximately 81% lower risk of suffering from depression than those who did not [51].

The structural model in our study confirmed an important role of the physical activity level as predictor of the perceived physical health, but not of the mental health, while MET measured as score did not result a predictor.

Despite most participants were overweight/obese, they showed on average a good physical activity level with mean total MET over the average cut-off. This can be considered a good result since higher levels of physical activity have been shown to be linked to lower motor decline in the elderly, while sedentary behavior was associated with impaired physical function [52]. Moreover, BMI or weight status did not influence overall performances negatively in the fitness tests; only a slight significance was found in the bivariate correlation between BMI and back scratch test results, with each increasing unit of BMI determining a score of 0.21 points lower. BMI and weight status weren’t even predictors of both physical and mental health, and this was also confirmed in the structural model.

Other correlations were evident for physical activity level and all fitness abilities, with the exception of back scratch test. This result could be explained by the type of physical activity that was performed by the study participants. In fact, most of them were engaged in walking or daily activity work such as gardening that did not involve the scapulohumeral joint.

Total MET score significantly predicted the upper limb strength and aerobic resistance, while a significant trend was showed for flexibility of the lumbar and ischio-crural musculature, agility and dynamic balance. A review by Lam et al. partially confirmed our results stating that higher levels of physical activity were correlated with higher levels of flexibility and balance [53].

Correlations for age in the bivariate model showed that perceived physical health decreased with aging, and this relationship is mediated by the abilities in some fitness tests. For example, we found that participants’ age was correlated with timed up and go test and arm curl test. In fact, a higher age corresponded to more time to perform the timed up and go test, and a higher age was correlated with lower strength as the repetitions of the arm curl were lower. These findings are consistent with studies by Cadore et al. [54] who show that as age increases there is a decrease in balance and gait speed. Other studies, confirming our findings, show that as age increases there is a decrease in balance, gait speed and a decrease in strength ratings [55, 56]. Age, however, wasn’t a predictor of the perceived physical and mental health in the structural model, and this is confirmed in another study where regression models were applied in a sample of European older adults [57].

Gender was correlated to sit and reach test, back scratch and minute step tests, with females performing overall better than males. A trend of significance was also found out for females perceiving a worst mental health status than males [58]. In the structural model, anyway, gender didn’t result to be a mere predictor of the perceived physical and mental health.

Our results showed even no correlation between perceived physical and mental health and adherence to the MD. This result appears opposite to the results found by Zaragoza Marti et al. [59] who found that adherence to the MD is directly associated with self-perceived physical and mental function. Our result, on the other hand, can be justified since on average the adherence of our group was below the limit of acceptability (MD score 8.4).

Since different papers confirm the lack of associations between mental health and variables such as age, gender, weight status and diet, focusing more on the psychological aspects such as depression or loneliness that predict mental health, the authors can suppose that the role of these psychological aspects could be stronger and there could be other psychological mediation mechanisms or neuro-metabolic explanations, that are age- and gender-independent or diet-independent, for these relationships.

### Limits and strengths

The study has some limitations. Firstly, our sample consisted almost entirely of women (88%) compared to men, so it was difficult to identify differences between genders. Therefore, it is worthwhile to undertake further research that includes a fair number of older men through effective recruitment methods targeted specifically at men.

Another limitation could be the use of the IPAQ questionnaire as the only method of measuring physical activity level in the elderly. Although the IPAQ is an internationally recognized questionnaire, whose validity and reliability were assessed in comparison to accelerometer, there are some studies that show some limitations of its use in the elderly population. The study by Tomioka et al. [60] conducted in elderly people over 65 shows that reliability was insufficient, while validity was adequate. Other studies indicate that the elderly underestimated both physical activity levels (moderate to vigorous physical activities and total physical activity) and sedentary behavior [61, 62]. A recent Italian study [63] proposed a modified version for the elderly population with acceptable consistency and reliability. But since it has not yet been published during our work, this is proposed to be used in future studies.

Our study has various strength points. Firstly, the fitness tests administered were well established and recognized, with the battery in the short form being valid and reliable for older populations. In addition, our sample was recruited from different living areas in the province of Palermo, these allowing comparisons based also on the urban or rural geographical characteristics of the sample.

The results of our research can also be applied to the individual because the selection criteria for participants and the evaluation methods used have been defined in detail. Finally, our study provides an important and original contribution to the knowledge of the relationship between perceived mental health and functional fitness in older adults, since there is a lack of this matter in the scientific literature.

## Conclusion

Referring to our results, the designed structural model was robust to describe predictors of perceived physical and mental health in older adults, which were recognized in the functional fitness both for the physical and mental health, and in the physical activity level for the perceived physical health.

Therefore, it would be strongly recommended for elderly subjects to engage in physical activity specifically targeted to older age, in order to improve fitness abilities.

Our results may be useful to clinicians and healthcare services for detecting and treating disease or its complications at an early stage, before symptoms or functional losses occur, thereby minimizing morbidity and mortality. In addition, they may be used to plan targeted strategies for promoting healthy lifestyle in elderly.

## References

[1] Peters E, Pritzkuleit R, Beske F, Katalinic A. [Demographic change and disease rates: a projection until 2050]. Bundesgesundheitsblatt, Gesundheitsforschung, Gesundheitsschutz. 2010;53(5):417–26. Epub 2010/04/09. doi: 10.1007/s00103-010-1050-y . 20376420

[2] WHO. Ageing and health 2022. https://www.who.int/news-room/fact-sheets/detail/ageing-and-health.

[3] Organization WH. Global action plan on physical activity 2018–2030: more active people for a healthier world. 2018.

[4] Rudnicka E, Napierała P, Podfigurna A, Męczekalski B, Smolarczyk R, Grymowicz M. The World Health Organization (WHO) approach to healthy ageing. Maturitas. 2020;139:6–11. Epub 2020/08/05. doi: 10.1016/j.maturitas.2020.05.018 . 32747042 PMC7250103

[5] Fulop T, Larbi A, Khalil A, Cohen AA, Witkowski JM. Are We Ill Because We Age? Frontiers in physiology. 2019;10:1508. Epub 2020/01/21. doi: 10.3389/fphys.2019.01508 . 31956310 PMC6951428

[6] Rikli RE, Jones CJ. Assessing Physical Performance in Independent Older Adults: Issues and Guidelines. Journal of Aging and Physical Activity. 1997;5(3):244–61. doi: 10.1123/japa.5.3.244

[7] Rikli RE, Jones CJ. Functional Fitness Normative Scores for Community-Residing Older Adults, Ages 60–94. Journal of Aging and Physical Activity. 1999;7:162–81.

[8] de Oliveira L, Souza EC, Rodrigues RAS, Fett CA, Piva AB. The effects of physical activity on anxiety, depression, and quality of life in elderly people living in the community. Trends in psychiatry and psychotherapy. 2019;41(1):36–42. Epub 2019/04/18. doi: 10.1590/2237-6089-2017-0129 . 30994779

[9] Andreescu C, Lee S. Anxiety Disorders in the Elderly. Advances in experimental medicine and biology. 2020;1191:561–76. Epub 2020/02/01. doi: 10.1007/978-981-32-9705-0_28 . 32002946

[10] Ohrnberger J, Fichera E, Sutton M. The dynamics of physical and mental health in the older population. Journal of the economics of ageing. 2017;9:52–62. Epub 2017/06/06. doi: 10.1016/j.jeoa.2016.07.002 . 28580276 PMC5446314

[11] Luo MS, Chui EWT, Li LW. The Longitudinal Associations between Physical Health and Mental Health among Older Adults. Aging & mental health. 2020;24(12):1990–8. Epub 2019/08/21. doi: 10.1080/13607863.2019.1655706 . 31429303

[12] An HY, Chen W, Wang CW, Yang HF, Huang WT, Fan SY. The Relationships between Physical Activity and Life Satisfaction and Happiness among Young, Middle-Aged, and Older Adults. International journal of environmental research and public health. 2020;17(13). Epub 2020/07/09. doi: 10.3390/ijerph17134817 . 32635457 PMC7369812

[13] Ord AS, Slogar SM, Sautter SW. Lifestyle Factors, Cognitive Functioning, and Functional Capacity in Older Adults. International journal of aging & human development. 2022;94(4):387–414. Epub 2021/04/30. doi: 10.1177/00914150211009467 . 33913787

[14] Seah B, Kowitlawakul Y, Jiang Y, Ang E, Chokkanathan S, Wang W. A review on healthy ageing interventions addressing physical, mental and social health of independent community-dwelling older adults. Geriatric nursing (New York, NY). 2019;40(1):37–50. Epub 2018/06/18. doi: 10.1016/j.gerinurse.2018.06.002 . 29909022

[15] Cunningham C, OS R, Caserotti P, Tully MA. Consequences of physical inactivity in older adults: A systematic review of reviews and meta-analyses. Scandinavian journal of medicine & science in sports. 2020;30(5):816–27. Epub 2020/02/06. doi: 10.1111/sms.13616 . 32020713

[16] Schuch FB, Vancampfort D, Richards J, Rosenbaum S, Ward PB, Stubbs B. Exercise as a treatment for depression: A meta-analysis adjusting for publication bias. Journal of psychiatric research. 2016;77:42–51. Epub 2016/03/16. doi: 10.1016/j.jpsychires.2016.02.023 . 26978184

[17] Livingston G, Sommerlad A, Orgeta V, Costafreda SG, Huntley J, Ames D, et al. Dementia prevention, intervention, and care. Lancet (London, England). 2017;390(10113):2673–734. Epub 2017/07/25. doi: 10.1016/S0140-6736(17)31363-6 . 28735855

[18] Govindaraju T, Sahle BW, McCaffrey TA, McNeil JJ, Owen AJ. Dietary Patterns and Quality of Life in Older Adults: A Systematic Review. Nutrients. 2018;10(8). Epub 2018/07/28. doi: 10.3390/nu10080971 . 30050006 PMC6115962

[19] Mazza E, Ferro Y, Pujia R, Mare R, Maurotti S, Montalcini T, et al. Mediterranean Diet In Healthy Aging. The journal of nutrition, health & aging. 2021;25(9):1076–83. Epub 2021/11/03. doi: 10.1007/s12603-021-1675-6 . 34725664 PMC8442641

[20] Coelho-Júnior HJ, Trichopoulou A, Panza F. Cross-sectional and longitudinal associations between adherence to Mediterranean diet with physical performance and cognitive function in older adults: A systematic review and meta-analysis. Ageing research reviews. 2021;70:101395. Epub 2021/06/22. doi: 10.1016/j.arr.2021.101395 . 34153553

[21] Knapik A, Brzęk A, Famuła-Wąż A, Gallert-Kopyto W, Szydłak D, Marcisz C, et al. The relationship between physical fitness and health self-assessment in elderly. Medicine. 2019;98(25):e15984. Epub 2019/06/25. doi: 10.1097/MD.0000000000015984 . 31232930 PMC6636929

[22] Perez FP, Perez CA, Chumbiauca MN. Insights into the Social Determinants of Health in Older Adults. Journal of biomedical science and engineering. 2022;15(11):261–8. Epub 2022/11/25. doi: 10.4236/jbise.2022.1511023 . 36419938 PMC9681180

[23] Ohrnberger J, Fichera E, Sutton M. The relationship between physical and mental health: A mediation analysis. Social science & medicine (1982). 2017;195:42–9. Epub 2017/11/14. doi: 10.1016/j.socscimed.2017.11.008 . 29132081

[24] Ullman JB. Structural equation modeling: reviewing the basics and moving forward. Journal of personality assessment. 2006;87(1):35–50. Epub 2006/07/22. doi: 10.1207/s15327752jpa8701_03 . 16856785

[25] Hurley AE, Scandura TA, Schriesheim CA, Brannick MT, Seers A, Vandenberg RJ, et al. Exploratory and confirmatory factor analysis: Guidelines, issues, and alternatives. Journal of Organizational Behavior. 1997;18(6):667–83. doi: 10.1002/(SICI)1099-1379(199711)18:6&lt;667::AID-JOB874&gt;3.0.CO;2-T

[26] Cole TJ, Bellizzi MC, Flegal KM, Dietz WH. Establishing a standard definition for child overweight and obesity worldwide: international survey. BMJ (Clinical research ed). 2000;320(7244):1240–3. Epub 2000/05/08. doi: 10.1136/bmj.320.7244.1240 . 10797032 PMC27365

[27] Ware J Jr., Kosinski M, Keller SD. A 12-Item Short-Form Health Survey: construction of scales and preliminary tests of reliability and validity. Medical care. 1996;34(3):220–33. Epub 1996/03/01. doi: 10.1097/00005650-199603000-00003 . 8628042

[28] Jakobsson U. Using the 12-item Short Form health survey (SF-12) to measure quality of life among older people. Aging clinical and experimental research. 2007;19(6):457–64. Epub 2008/01/04. doi: 10.1007/BF03324731 . 18172367

[29] Bentur N, King Y. The challenge of validating SF-12 for its use with community-dwelling elderly in Israel. Quality of life research: an international journal of quality of life aspects of treatment, care and rehabilitation. 2010;19(1):91–5. Epub 2009/12/17. doi: 10.1007/s11136-009-9562-3 . 20012210

[30] Ottoboni G, Cherici A, Marzocchi M, Chattat R. Algoritimi di calcolo per gli indici PCS e MCS del questinario SF-122017.

[31] Rikli RE, Jones CJ. Senior fitness test manual: Human kinetics; 2013.

[32] Rikli R, Jones J. Development and validation of a functional fitness test for a community-residing adults. Journal of Aging and Physical Activity. 1999;7:129–61. doi: 10.1123/japa.7.2.129

[33] Bisciotti GN. L’invecchiamento. Biologia, fisiologia e strategie anti-aging2013 1 Gennaio 2013. 231 p.

[34] Langhammer B, Stanghelle JK. The Senior Fitness Test. Journal of physiotherapy. 2015;61(3):163. Epub 2015/06/06. doi: 10.1016/j.jphys.2015.04.001 . 26044346

[35] Craig CL, Marshall AL, Sjöström M, Bauman AE, Booth ML, Ainsworth BE, et al. International physical activity questionnaire: 12-country reliability and validity. Medicine and science in sports and exercise. 2003;35(8):1381–95. Epub 2003/08/06. doi: 10.1249/01.MSS.0000078924.61453.FB . 12900694

[36] Wolin KY, Heil DP, Askew S, Matthews CE, Bennett GG. Validation of the International Physical Activity Questionnaire-Short among Blacks. Journal of physical activity & health. 2008;5(5):746–60. Epub 2008/09/30. doi: 10.1123/jpah.5.5.746 . 18820348 PMC2744347

[37] Bertocchi L, Vecchio R, Sorbello S, Correale L, Gentile L, Buzzachera C, et al. Impact of the COVID-19 pandemic on physical activity among university students in Pavia, Northern Italy. Acta bio-medica: Atenei Parmensis. 2021;92(S6):e2021443. Epub 2021/12/11. doi: 10.23750/abm.v92iS6.12232 . 34889314 PMC8851014

[38] Abate Daga F, Agostino S, Peretti S, Beratto L. COVID-19 nationwide lockdown and physical activity profiles among North-western Italian population using the International Physical Activity Questionnaire (IPAQ). Sport sciences for health. 2021;17(2):459–64. Epub 2021/03/11. doi: 10.1007/s11332-021-00745-8 . 33688376 PMC7931493

[39] Bekar C, Goktas Z. Validation of the 14-item mediterranean diet adherence screener. Clinical nutrition ESPEN. 2023;53:238–43. Epub 2023/01/20. doi: 10.1016/j.clnesp.2022.12.026 . 36657918

[40] García-Conesa MT, Philippou E, Pafilas C, Massaro M, Quarta S, Andrade V, et al. Exploring the Validity of the 14-Item Mediterranean Diet Adherence Screener (MEDAS): A Cross-National Study in Seven European Countries around the Mediterranean Region. Nutrients. 2020;12(10). Epub 2020/10/01. doi: 10.3390/nu12102960 . 32992649 PMC7601687

[41] Fontalba-Romero MI, Lopez-Enriquez S, Lago-Sampedro A, García-Escobar E, Pastori RL, Domínguez-Bendala J, et al. Association between the Mediterranean Diet and Metabolic Syndrome with Serum Levels of miRNA in Morbid Obesity. Nutrients. 2021;13(2). Epub 2021/02/13. doi: 10.3390/nu13020436 . 33572759 PMC7911421

[42] Schröder H, Fitó M, Estruch R, Martínez-González MA, Corella D, Salas-Salvadó J, et al. A short screener is valid for assessing Mediterranean diet adherence among older Spanish men and women. The Journal of nutrition. 2011;141(6):1140–5. Epub 2011/04/22. doi: 10.3945/jn.110.135566 . 21508208

[43] Kline RB. Principles and practice of structural equation modeling, 4th ed. New York, NY, US: Guilford Press; 2016. xvii, 534–xvii, p.

[44] Buchman AS, Boyle PA, Wilson RS, Bienias JL, Bennett DA. Physical activity and motor decline in older persons. Muscle & nerve. 2007;35(3):354–62. Epub 2006/12/05. doi: 10.1002/mus.20702 . 17143881

[45] Stathokostas L, McDonald MW, Little RM, Paterson DH. Flexibility of older adults aged 55–86 years and the influence of physical activity. Journal of aging research. 2013;2013:743843. Epub 2013/07/19. doi: 10.1155/2013/743843 . 23862064 PMC3703899

[46] Walker JM, Sue D, Miles-Elkousy N, Ford G, Trevelyan H. Active mobility of the extremities in older subjects. Physical therapy. 1984;64(6):919–23. Epub 1984/06/01. doi: 10.1093/ptj/64.6.919 . 6728913

[47] Miotto JM, Chodzko-Zajko W, Reich J, Supler M. Reliability and Validity of the Fullerton Functional Fitness Test: An Independent Replication Study. Journal of Aging and Physical Activity. 1999;7:339–53.

[48] Pucci GC, Rech CR, Fermino RC, Reis RS. Association between physical activity and quality of life in adults. Revista de saude publica. 2012;46(1):166–79. Epub 2012/01/18. doi: 10.1590/s0034-89102012000100021 . 22249758

[49] Hemmeter UM, Ngamsri T. [Physical Activity and Mental Health in the Elderly]. Praxis. 2022;110(4):193–8. Epub 2022/03/17. doi: 10.1024/1661-8157/a003853 . 35291872

[50] Tesler R, Ihle A, Marques A. Editorial: Association of physical activity and fitness with mental health outcomes: Current advances and future directions. Frontiers in public health. 2022;10:1027395. Epub 2022/10/04. doi: 10.3389/fpubh.2022.1027395 . 36187652 PMC9521418

[51] Byeon H. Relationship between Physical Activity Level and Depression of Elderly People Living Alone. International journal of environmental research and public health. 2019;16(20). Epub 2019/10/28. doi: 10.3390/ijerph16204051 . 31652619 PMC6843978

[52] Garcia Meneguci CA, Meneguci J, Sasaki JE, Tribess S, Júnior JSV. Physical activity, sedentary behavior and functionality in older adults: A cross-sectional path analysis. PloS one. 2021;16(1):e0246275. Epub 2021/01/30. doi: 10.1371/journal.pone.0246275 . 33513196 PMC7846014

[53] Lam FM, Huang MZ, Liao LR, Chung RC, Kwok TC, Pang MY. Physical exercise improves strength, balance, mobility, and endurance in people with cognitive impairment and dementia: a systematic review. Journal of physiotherapy. 2018;64(1):4–15. Epub 2018/01/01. doi: 10.1016/j.jphys.2017.12.001 . 29289581

[54] Cadore EL, Rodríguez-Mañas L, Sinclair A, Izquierdo M. Effects of different exercise interventions on risk of falls, gait ability, and balance in physically frail older adults: a systematic review. Rejuvenation research. 2013;16(2):105–14. Epub 2013/01/19. doi: 10.1089/rej.2012.1397 . 23327448 PMC3634155

[55] Mitchell WK, Williams J, Atherton P, Larvin M, Lund J, Narici M. Sarcopenia, dynapenia, and the impact of advancing age on human skeletal muscle size and strength; a quantitative review. Frontiers in physiology. 2012;3:260. Epub 2012/08/31. doi: 10.3389/fphys.2012.00260 . 22934016 PMC3429036

[56] Walston JD. Sarcopenia in older adults. Current opinion in rheumatology. 2012;24(6):623–7. Epub 2012/09/08. doi: 10.1097/BOR.0b013e328358d59b . 22955023 PMC4066461

[57] König H-H, Heider D, Lehnert T, Riedel-Heller SG, Angermeyer MC, Matschinger H, et al. Health status of the advanced elderly in six European countries: results from a representative survey using EQ-5D and SF-12. Health and quality of life outcomes. 2010;8(1):1–11. doi: 10.1186/1477-7525-8-143 21114833 PMC3009699

[58] Guízar-Sánchez D, Yoldi-Negrete M, Robles-García R, López-Ortiz G, Rivero-López C, Castro-Valdes I, et al. Self-Perceived Mental Health and Perceived Discrimination in Family Physicians and Residents: A Comparative Study Between Men and Women. Journal of the American Board of Family Medicine: JABFM. 2022;35(5):912–20. Epub 2022/10/19. doi: 10.3122/jabfm.2022.05.220091 . 36257698

[59] Zaragoza-Martí A, Cabañero-Martínez MJ, Hurtado-Sánchez JA, Laguna-Pérez A, Ferrer-Cascales R. Evaluation of Mediterranean diet adherence scores: a systematic review. BMJ open. 2018;8(2):e019033. Epub 2018/02/27. doi: 10.1136/bmjopen-2017-019033 . 29478018 PMC5855302

[60] Tomioka K, Iwamoto J, Saeki K, Okamoto N. Reliability and validity of the International Physical Activity Questionnaire (IPAQ) in elderly adults: the Fujiwara-kyo Study. Journal of epidemiology. 2011;21(6):459–65. Epub 2011/09/29. doi: 10.2188/jea.je20110003 . 21946625 PMC3899462

[61] Cleland C, Ferguson S, Ellis G, Hunter RF. Validity of the International Physical Activity Questionnaire (IPAQ) for assessing moderate-to-vigorous physical activity and sedentary behaviour of older adults in the United Kingdom. BMC medical research methodology. 2018;18(1):176. Epub 2018/12/24. doi: 10.1186/s12874-018-0642-3 . 30577770 PMC6303992

[62] Van Holle V, De Bourdeaudhuij I, Deforche B, Van Cauwenberg J, Van Dyck D. Assessment of physical activity in older Belgian adults: validity and reliability of an adapted interview version of the long International Physical Activity Questionnaire (IPAQ-L). BMC public health. 2015;15:433. Epub 2015/05/01. doi: 10.1186/s12889-015-1785-3 . 25928561 PMC4427934

[63] Iona T, Masala D, La Torre G, Imbrogna A, Mannocci A. International Physical Activity Questionnaire for ITalian Elderly (IPAQ-EIT): reliability in an Italian sample. La Clinica terapeutica. 2022;173(6):546–50. Epub 2022/11/15. doi: 10.7417/CT.2022.2480 . 36373453

